# An Improved Perry–Robertson Theory for Buckling Prediction of Unidirectional-Fiber-Reinforced Composite Insulators

**DOI:** 10.3390/ma19132876

**Published:** 2026-07-05

**Authors:** Yandong Shi, Wenkai Li, Xuming Su, Linjun Zhang

**Affiliations:** 1 Zhejiang Provincial Engineering Center of Integrated Manufacturing Technology and Intelligent Equipment, Hangzhou City University, Hangzhou 310015, China; shiyd@hzcu.edu.cn (Y.S.); xumgsu@yahoo.com (X.S.); 2 Key Laboratory of Composite Materials on Electrical Insulation, National Energy Administration, Jiangsu Shemar Electric Co., Ltd., Nantong 226017, China; zljun@shenmapower.com

**Keywords:** composite insulators, glass fiber reinforced polymer, Perry–Robertson theory, buckling, nonlinear material behavior

## Abstract

Unidirectional glass fiber reinforced polymer (GFRP) composite insulators are widely used in extra-high voltage (EHV) and ultra-high voltage (UHV) transmission lines due to their outstanding electrical and mechanical performance. However, the accurate prediction of the critical buckling load is crucial to satisfy the high reliability requirement under complex operations. In this paper, an improved Perry–Robertson theory to predict the critical buckling loads of GFRP composite insulators with different slenderness is proposed. Firstly, initial imperfection is expressed as a function of the insulator strut length, which enables the critical load to be formulated as a function of slenderness explicitly. It also allows for convenient comparisons with other theories, such as Euler and Johnson’s, and easy calibration with the magnitude of initial imperfections. Secondly, the nonlinear material behavior of the GFRP composite insulator strut, resulting from changes in glass fiber orientation in relation to the loading direction during buckling, is considered to further enhance the prediction accuracy. The predicted results with current theory were validated through compression tests of GFRP composite insulators with solid and hollow struts and different slenderness and boundary conditions, which shows an accuracy of over 85%. Thus, the proposed improved Perry–Robertson theory can be also applied in other fiber-reinforced composite buckling analyses.

## 1. Introduction

Compared to traditional electricity power transmission towers that use all-steel structures with porcelain or glass insulators, power transmission towers that utilize polymer composite insulators offer several advantages, including excellent anti-pollution flashover performance, reduced size, lighter weight, and extremely high mechanical strength. Furthermore, they require zero inspection exemption and less maintenance and offer convenient transportation [1,2,3]. Despite these benefits, the wider application of polymer composites in power-transmission facilities is still hindered by high production and manufacturing costs, as well as excessive design margins that need to be addressed.

Most polymer composite insulators used for tower and other structures in power grip are pultruded glass fiber reinforced polymer (GFRP) composites. During the pultrusion process, fibers, wetted by going through a resin pool, are pulled through a heated die that provides the required cross-section to the final product. As a result, the fibers in the pultruded GFRP composite insulators are considered unidirectional and uniform along the length direction. Since axial compressive load is the most common operation condition for GFRP insulator struts, the prediction of buckling load is of great importance. The buckling behavior of a unidirectional fiber-reinforced polymer composite has been extensively investigated in prior state-of-arts. Tomblin [4] has studied the local buckling mechanism of thin FRP pultrusion column with a solid section and proposes a new method to solve the local buckling load based on the Southwell method, which can consider the slight deviation frequently found in pultrusion manufacturing well. Zureik [5] has proposed a modified Euler formula considering shear deformation. However, based on Roberts’s [6] theoretical study on the effect of shear deformation on the flexural and torsional buckling of pultrusion-fiber-reinforced composites, it is found that the contribution of shear action to the stability of composite components is not significant, generally less than 5%.

In the existing research on the critical buckling-load-prediction problem of composite axial compression stability, the critical buckling load is mostly predicted using a theoretical model formula and the finite element method (FEM). The theoretical formula involves the Euler formula, Johnson formula, and Perry–Robertson formula. Hashem and Yuan [7] respectively have carried out axial compression tests on medium- and short-length GFRP columns with slenderness ratios varying from 0.5 to 1.5 and used the Euler formula to calculate the critical buckling load of medium-length columns. However, due to the initial defects of actual axial compression members and the anisotropy of composite materials, the ultimate bearing capacity of the composite insulating pipe cannot be simply calculated using Euler’s formula [8,9]. Johnson’s formula and Perry’s formula are two well-known modifications of Euler’s formula that are the basis of some design criteria. The Johnson formula is an interpolation between the yield stress of the column material and the critical stress given by the Euler formula. The formula is developed based on the experimental results of Johnson in around 1900 [10]. The Perry–Robertson formula is a mathematical formula that can approximate the buckling load of long and thin pillars well [11], and it is the basis of the buckling formula adopted by EN 1993 [12]. Xue et al. [13] carried out stability tests on epoxy resin matrix composite tubes with various slenderness ratios and compared the experimental results with the predictions of the classical Perry–Robertson formula. They reported that the maximum prediction error reached 48.66%. Such discrepancies may arise because the classical formulation assumes a constant elastic modulus and employs an implicit imperfection parameter, whereas the stiffness degradation associated with material anisotropy and fiber misalignment, as well as the effect of geometric imperfections, are not explicitly considered. These factors become particularly important in the intermediate slenderness range, leading to reduced prediction accuracy. These observations provide part of the motivation for the present study. The finite element method (FEM) is also one of the commonly used methods to calculate the critical buckling load under axial compressive instability. By discretizing and approximating the structure, this method transforms the FEM into a generalized eigenvalue problem of matrix [14], but the solving accuracy depends on the mesh size and element shape function. Zienkiewicz [15] established eigenvalue equations through a geometric stiffness matrix and element stiffness matrix and then realized the solving of generalized eigenvalue problems to determine buckling loads. Chen [16] established the flexibility equation of the member after buckling, worked out the inverse of the flexibility equation to obtain the element stiffness equation, and finally determined the buckling load of the structure by solving the transcendent equation. Eisenberger [17] constructed an approximately exact finite element for the axial buckling analysis by means of power-level digital migration of differential governing equations, but this method is not easy to achieve when solving buckling loads with finite element software by dispersing and approximating structures.

In recent years, considerable efforts have been devoted to improving the prediction accuracy of pultruded FRP columns through experimental, numerical, and analytical approaches. Experimental and numerical studies have been conducted on pultruded GFRP tubular and channel columns under various loading conditions, revealing the strong influence of the slenderness, cross-sectional configuration, and material orthotropy on the failure mode and buckling capacity [18,19,20]. More recently, the local–global interaction and the effects of multidirectional fiber architectures have also been investigated [21]. Comprehensive reviews have summarized the existing prediction methods and highlighted the continuing development of Euler-based, shear-corrected, Southwell, Perry-type, and machine-learning-based formulations for pultruded FRP members [22]. In addition, data-driven approaches based on machine learning have been proposed to improve the prediction accuracy by utilizing large experimental databases [23].

The aforementioned studies indicate that existing analytical models, including the Euler, Johnson, and classical Perry–Robertson formulations, can provide satisfactory predictions for ideal columns and metallic structures. However, their prediction accuracy may deteriorate when applied to pultruded GFRP members with manufacturing imperfections and moderate-to-high slenderness ratios. In particular, the effects of geometric imperfections and stiffness variation associated with material anisotropy are not explicitly incorporated in the classical formulations, which may lead to noticeable discrepancies between theoretical predictions and experimental results. In addition, although the FEM can provide accurate predictions, it generally requires detailed material characterization and relatively high computational cost, limiting its convenience for engineering design and rapid assessments. Therefore, there is still a need for an analytical formulation that can explicitly account for geometric imperfections and the stiffness variation associated with fiber misalignment while maintaining computational efficiency. In this paper, the stability test of a glass-fiber-reinforced epoxy resin matrix composite was carried out. An improved Perry–Robertson formula considering the initial defects of the member was proposed to predict the critical buckling load, and the relationship between the initial defects of the cylinder and the critical load of instability was established. Additionally, the effect of the anisotropy of the elastic properties of the composite material was considered, as there is a certain angle deflection between the local direction of the fiber and the loading direction under axial compression load. The nonlinear problem of the bending stiffness of the composite material under compressive load was also taken into account. Through axial compression instability tests on four composite insulator components with different slenderness ratio specifications, it was observed that the prediction accuracy of the model established in this paper is over 85% based on the comparison and analysis of the test results and theoretical results.

## 2. A New Expression of Perry–Robertson’s Buckling Theory

**Figure 1:**
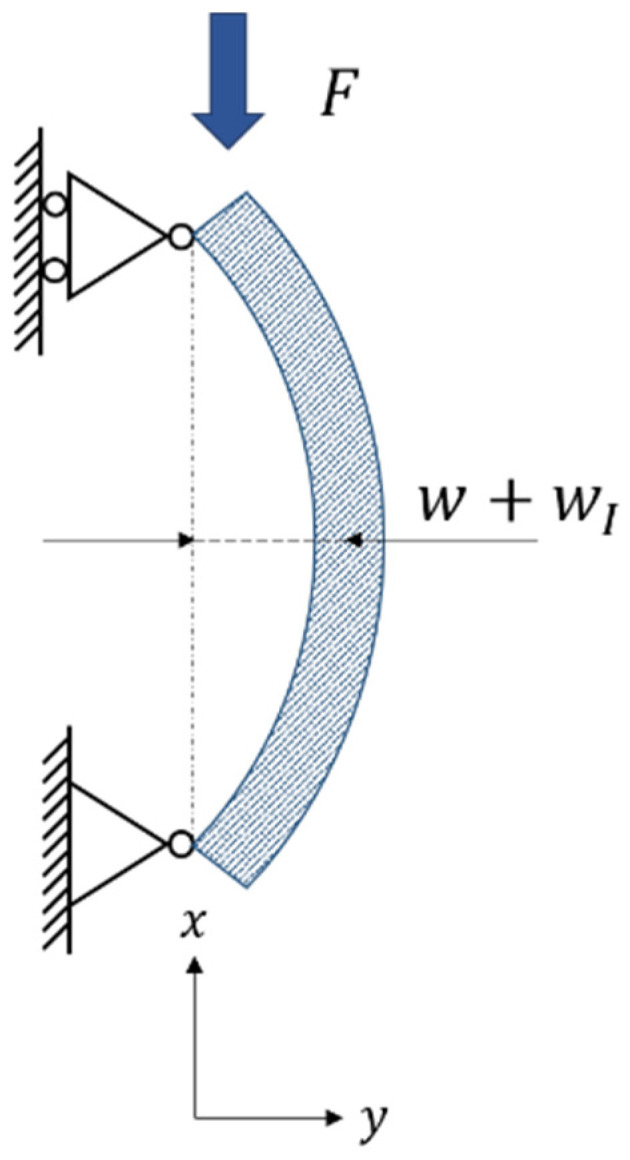
A simply supported strut.

For a simply supported strut with a length *L* as shown in Figure 1, the equilibrium equation is [24]

(1) $$EI\frac{d^{2}w}{{dx}^{2}}=M=-P(w+w_{I})\;$$

where *M* is the sectional moment, *w* is deflection in the *y* direction, *E* is the material Young’s modulus, and *I* is the sectional moment of inertial. $w_{I}$ is the initial imperfection of the strut.

The boundary conditions of the strut are

(2) $$w=0\;and\;\frac{d^{2}w}{{dx}^{2}}=\;0\;at\;x\;=\;0\;and\;x\;=\;L$$

It is well known the eigenfunctions of Equations (1) and (2) are sinusoidal functions. The initial imperfection of the strut is thus written as

(3) $$w_{I}=\omega_{I}sin(\frac{\pi x}{L})$$

where $\omega_{I}$ is the deviation of the strut in the middle ($x=\frac{L}{2}$) from the supposed straight line connecting two ends of the strut and is related to manufacturing processes. Equation (1) with boundary conditions (2) and initial imperfection (3) has the solution

(4) $$w=\frac{\omega_{I}}{\frac{EI\pi^{2}}{PL^{2}}-1}sin(\frac{\pi x}{L})$$

Apparently when

(5) $$P=P_{e}=\frac{EI\pi^{2}}{L^{2}}$$

the defection of strut goes infinite, and the strut fails. $P_{e}$ is the classical Euler’s buckling failure load, which can be written in terms of the slenderness of a strut

(6) $$P_{e}=\frac{EI\pi^{2}}{L^{2}}=\frac{E\pi^{2}Ar^{2}}{L^{2}}=\frac{EA\pi^{2}}{\lambda^{2}}$$

where slenderness is defined as $\lambda =\frac{L}{r}$. And *r* is the gyration of the cross-section, related to the moment of inertia $I=Ar^{2}$.

Substituting Equation (6) into the definition $\sigma_{e}=\frac{P_{e}}{A}$, the Euler failure stress can be expressed as

(7) $$\sigma_{e}=\frac{P_{e}}{A}=\frac{E{A\pi }^{2}}{{A\lambda }^{2}}=\frac{E\pi^{2}}{\lambda^{2}}$$

For a strut with small slenderness, $\sigma_{e}$ can be easily larger than the material failure stress under compressive loading. The Perry–Robertson formulation, which is the basis for the buckling formulation adopted in EN 1993, assumes that a strut will fail when the maximum stress of the strut reaches the yield stress, or the ultimate compressive stress for the glass fiber composite. The stress of the strut consists of compressive stress and bending stress. The compressive stress is uniform throughout the strut, and the bending stress changes with the moment. The maximum stress of the simply supported strut occurs at the edge of the cross-section of the maximum moment. The maximum moment occurs at the middle of the strut and is

(8) $$M_{max}=M{|}_{x=\frac{L}{2}}={(\omega }_{I}+\frac{\omega_{I}P}{P_{e}-P})P=\frac{\omega_{I}P_{e}P}{P_{e}-P}$$

And the maximum stress is thus

(9) $$\sigma_{max}=\frac{P}{A}+\frac{M_{max}c}{I}=\frac{P}{A}\left(1+\frac{c}{r^{2}}\frac{\omega_{I}P_{e}}{P_{e}-P}\right)=\sigma (1+\frac{c\omega_{I}}{r^{2}}\frac{\sigma_{e}}{\sigma_{e}-\sigma })$$

According to the Perry–Robertson formulation, failure is assumed to occur when the maximum compressive stress reaches the ultimate compressive strength of the material, i.e., $\sigma_{max}=\sigma_{u}$. Substituting Equation (9) into the above condition gives

(10) $$\sigma_{u}=\sigma (1+\frac{c\omega_{I}}{r^{2}}\frac{\sigma_{e}}{\sigma_{e}-\sigma })$$

where *c* is the distance from the extreme edge of the strut to the neutral axis, and $\sigma_{u}$ the ultimate stress for compression. The solution to Equation (10) is [3]

(11) $$\sigma =\frac{\left({\sigma_{e}+\sigma }_{u}{+\frac{c\omega_{I}}{r^{2}}\sigma }_{e}\right)-\sqrt{{\left({\sigma_{e}+\sigma }_{u}{+\frac{c\omega_{I}}{r^{2}}\sigma }_{e}\right)}^{2}-4{\sigma_{e}\sigma }_{u}}}{2}$$

It is noted that both *c* and *r* in Equation (11) are geometrical parameters of the strut cross-section, and their ratio remains constant for a given cross-sectional shape. Therefore, c can be expressed as

(12) c=κr

where κ is a dimensionless parameter determined by the sectional geometry.

Initial geometric imperfections are commonly considered in stability analysis and are often assumed to increase with the member length. Consistent with the engineering approximations adopted in Eurocode 3 [12], GB50017 [25], and related studies [26], a first-order linear relationship is adopted in the present study, i.e.,

(13) $$\omega_{I}=kL$$

where $\omega_{I}$ is the amplitude of the initial imperfection and k is a dimensionless coefficient characterizing its magnitude. Although other functional forms may also be possible, the linear form provides a simple representation with only one calibration parameter and facilitates subsequent analytical derivations. Similar assumptions have been widely adopted in engineering stability analyses and have been shown to provide satisfactory predictions for practical applications.

**Figure 2:**
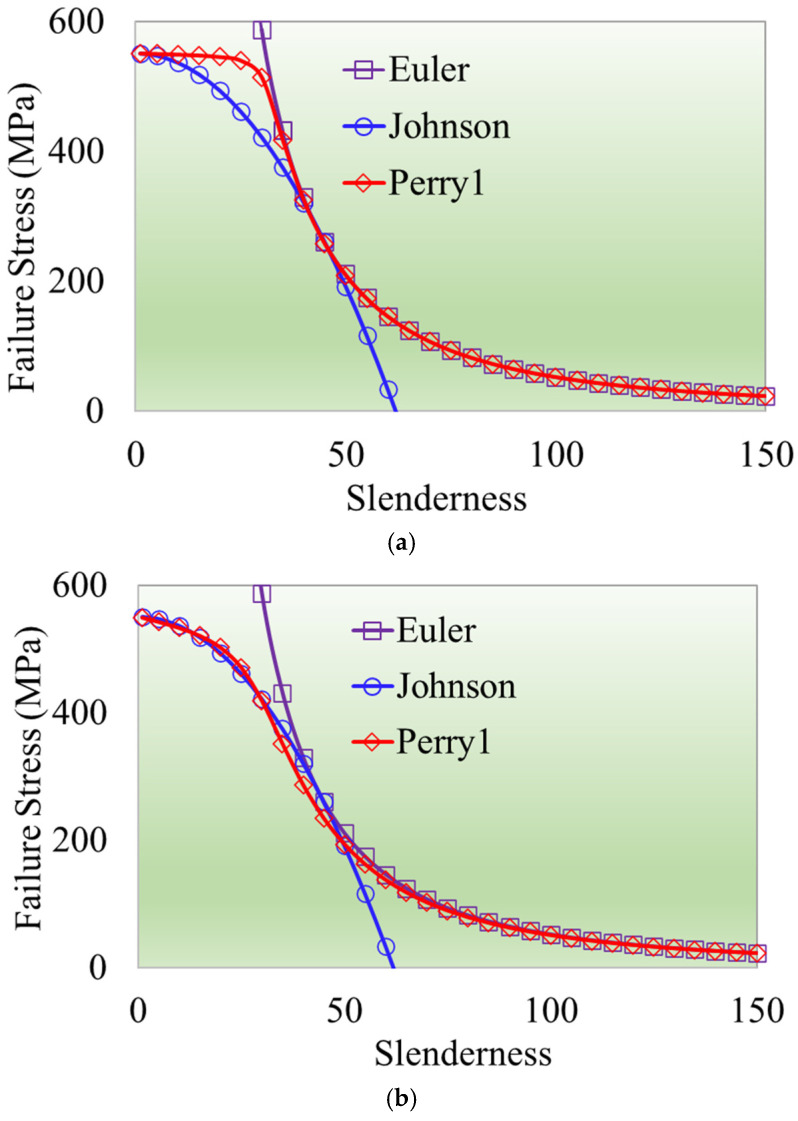
Comparison of failure stress of the new Perry–Robertson formulation with Euler and Johnson stress for case (**a**) ϵ=0.0003 and (**b**) ϵ=0.003. For both cases, E = 53.5 GPa, $\sigma_{u}=55.2\;$MPa.

Substituting Equations (12) and (13) into Equation (11) and reorganizing gives

(14) $$\sigma =\frac{\left({\sigma_{e}+\sigma }_{u}{+\epsilon \lambda \sigma }_{e}\right)-\sqrt{{\left({\sigma_{e}+\sigma }_{u}{+\lambda \epsilon \sigma }_{e}\right)}^{2}-4{\sigma_{e}\sigma }_{u}}}{2}$$

where ϵ=κk. Note that $\sigma_{e}=\frac{E\pi^{2}}{\lambda^{2}}$, and Equation (14) formulates Perry–Robertson’s failure stress explicitly as a function of the slenderness of the strut, with *k* as a parameter defining the magnitude of manufacturing imperfection. Equation (14) is a new expression and has the advantage of being able to easily study the impact of initial imperfection and slenderness and to compare with other failure criteria. Figure 2 shows failure stresses calculated by using Equation (14) for two different initial imperfections, i.e., ϵ=κk=0.0003 and ϵ=κk=0.003, respectively. Also in the figure are Euler failure stress and that of Johnson’s failure stress. Johnson’s parabolic formula interpolates between the yield stress (ultimate stress) of the material to Euler’s buckling stress by the slenderness ratio. Johnson’s approach has been adopted in many North American structural design codes in which a moderate amount of imperfection has been implicitly accounted for [7].

It is expected that the magnitude of initial imperfection has a profound influence on the failure stress. The Perry–Robertson failure stress is almost coinciding with that of Johnson’s when ϵ=0.003. For a circular strut, this amounts to k=0.0015 meaning that there is about 1.5 mm deviation at the center of the strut away from the straight line of a strut of length 1000 mm, corresponding to an imperfection-to-length ratio of 0.15%. It would be interesting to note that the Johnson failure formula is empirical in nature, while Equation (14) is, however, based on physically motivated assumptions.

## 3. A Nonlinear Material Version of Perry–Robertson’s Buckling Theory

The equilibrium Equation (1) assumes that Young’s modulus is constant throughout the length of the strut. For buckling analysis of a strut, the loading direction is assumed to be in the original strut-length direction. For unidirectional reinforced glass fiber composite, the fiber orientation deviates from the loading direction once the imperfection and deflection are introduced. Since the rotations involved are assumed to be small, the small-angle approximation is adopted, i.e., θ≈tanθ. And it is well known that mechanical properties of GFRP are highly fiber-orientation-dependent and Young’s modulus is a function of the loading angle to the fiber orientation, $\theta =\frac{d(w+w_{I})}{dx}$ [27]. As Young’s modulus is symmetric to *θ*, the first two orders of approximation of *E* in terms of θ are

(15) $$E=E_{0}-E_{a}[\theta {]}^{2}=E_{0}-E_{a}[\frac{d\left(w+w_{I}\right)}{dx}{]}^{2}$$

And Equation (1) turns into

(16) $$\{E_{0}-E_{a}[\frac{d(w+w_{I})}{dx}{]}^{2}\}I\frac{d^{2}w}{{dx}^{2}}+Pw=-Pw_{I}$$

Equation (16) is a nonlinear ordinary differential equation. For small strains and for an initially straight strut,

(17) $$w\sim w_{I}\sim w\prime \sim w_{I}\prime \sim w\prime \prime \sim w_{I}\prime \prime \ll 1$$

Under these assumptions, the fiber-misalignment angle remains small. Therefore, the expansion of E(θ) in Equation (15) is truncated at the leading nonlinear term $\theta^{2}$, while higher-order terms such as $\theta^{4}$ and above are neglected. Consequently, Equation (16) represents a weakly nonlinear differential equation. A perturbation analysis procedure can be carried out for an approximate solution of Equation (16). Higher-order terms in Equation (16) are first moved to the right,

(18) $$E_{0}I\frac{d^{2}w}{{dx}^{2}}+Pw=-Pw_{I}{+E}_{a}I[\frac{d(w+w_{I})}{dx}{]}^{2}\frac{d^{2}w}{{dx}^{2}}$$

The solution of Equation (18) is written in the form

(19) $$w=w_{1}+w_{2}+w_{3}\ldots$$

where

(20) $$w_{1}\sim w_{I}\gg w_{2}\gg w_{3}\ldots$$

Substituting Equation (19) into (18) and organizing the terms by its orders for approximation defined by Equation (20), one obtains

(21) $$\begin{array}{c} {(E}_{0}I\frac{d^{2}w_{1}}{{dx}^{2}}+Pw_{1})+{(E}_{0}I\frac{d^{2}w_{2}}{{dx}^{2}}+Pw_{2})+{(E}_{0}I\frac{d^{2}w_{3}}{{dx}^{2}}+Pw_{3})+\ldots =\\ -Pw_{I}{+E}_{a}I[\frac{d(w_{1}+w_{I})}{dx}{]}^{2}\frac{d^{2}w_{1}}{{dx}^{2}}{+E}_{a}I[2\frac{d{(w}_{1}+w_{I})}{dx}\frac{dw_{2}}{dx}\frac{d^{2}w_{1}}{{dx}^{2}}+(\frac{d{(w}_{1}+w_{I})}{dx}{)}^{2}\frac{d^{2}w_{2}}{{dx}^{2}}]\ldots \end{array}$$

This leads to a series of differential equations, and only the first three are to be solved. They are

(22) $$E_{0}I\frac{d^{2}w_{1}}{{dx}^{2}}+Pw_{1}=-Pw_{I}$$

(23) $$E_{0}I\frac{d^{2}w_{2}}{{dx}^{2}}+Pw_{2}=0$$

(24) $$E_{0}I\frac{d^{2}w_{3}}{{dx}^{2}}+Pw_{3}{=E}_{a}I[\frac{d(w_{1}+w_{I})}{dx}{]}^{2}\frac{d^{2}w_{1}}{{dx}^{2}}$$

…

The boundary conditions can be treated accordingly to lead to

(25) $$w_{i}=0\;and\;\frac{d^{2}w_{i}}{{dx}^{2}}=0\;for\;x=0,\;x=L\;and\;all\;i$$

The solution of Equations (22) and (25) is obtained as in the previous section and is

(26) $$w_{1}=\frac{\omega_{I}P}{P_{e}-P}sin(\frac{\pi x}{L})$$

The solution of Equation (23) is zero

(27) $$w_{2}=0$$

Substituting Equation (26) into the nonlinear forcing term in Equation (18), the right-hand side is proportional to $[\frac{d(w_{1}+w_{I})}{dx}{]}^{2}\frac{d^{2}w_{1}}{{dx}^{2}}$, which can be expressed as $[\frac{d(w_{1}+w_{I})}{dx}{]}^{2}\frac{d^{2}w_{1}}{{dx}^{2}}\propto sin\left(\frac{\pi x}{L}\right){cos}^{2}\left(\frac{\pi x}{L}\right)$. By applying the trigonometric identity, namely $sin\left(\frac{\pi x}{L}\right){cos}^{2}\left(\frac{\pi x}{L}\right)=\frac{1}{4}[sin\left(\frac{\pi x}{L}\right)+sin\left(\frac{3\pi x}{L}\right)]$, the nonlinear forcing term is decomposed into the first and third harmonic components. After collecting terms and substituting $P_{e}=\frac{EI\pi^{2}}{L^{2}}$, one obtains

(28) $$E_{0}I\frac{d^{2}w_{3}}{{dx}^{2}}+Pw_{3}{=-\frac{1}{4}E}_{a}I(\frac{\pi }{L}{)}^{4}(\frac{\omega_{I}P_{e}}{P_{e}-P}{)}^{3}(\frac{P}{P_{e}})[sin\left(\frac{\pi x}{L}\right)+sin\left(\frac{3\pi x}{L}\right)]$$

The solution of Equation (28) with an appropriate boundary condition in Equation (25) is

(29) $$w_{3}=\frac{{\frac{1}{4}E}_{a}I{\left(\frac{w_{I}P_{e}}{P_{e}-P}\right)}^{3}{(\frac{P}{P_{e}})\left(\frac{\pi }{L}\right)}^{4}}{P_{e}-P}sin\left(\frac{\pi x}{L}\right)+\frac{{\frac{1}{4}E}_{a}I{\left(\frac{w_{I}P_{e}}{P_{e}-P}\right)}^{3}{(\frac{P}{P_{e}})\left(\frac{\pi }{L}\right)}^{4}}{9P_{e}-P}sin\left(\frac{3\pi x}{L}\right)$$

And thus

(30) $$\begin{array}{c} {w=w_{1}+w}_{2}+w_{3}+higher\;order\;terms\;\left(h.o.t.\right)=\frac{w_{I}P}{P_{e}-P}sin\left(\frac{\pi x}{L}\right)+\\ \frac{{\frac{1}{4}E}_{a}I{\left(\frac{w_{I}P_{e}}{P_{e}-P}\right)}^{3}(\frac{P}{P_{e}}){\left(\frac{\pi }{L}\right)}^{4}}{P_{e}-P}sin\left(\frac{\pi x}{L}\right)+\frac{{\frac{1}{4}E}_{a}I{\left(\frac{w_{I}P_{e}}{P_{e}-P}\right)}^{3}(\frac{P}{P_{e}}){\left(\frac{\pi }{L}\right)}^{4}}{9P_{e}-P}sin\left(\frac{3\pi x}{L}\right)+h.\;o.\;t. \end{array}$$

The maximum deflection occurs at *x = L*/2, and

(31) $$w_{max}=\frac{w_{I}P}{P_{e}-P}+\frac{{\frac{1}{4}E}_{a}I{\left(\frac{w_{I}P_{e}}{P_{e}-P}\right)}^{3}(\frac{P}{P_{e}}){\left(\frac{\pi }{L}\right)}^{4}}{P_{e}-P}+\frac{{\frac{1}{4}E}_{a}I{\left(\frac{w_{I}P_{e}}{P_{e}-P}\right)}^{3}(\frac{P}{P_{e}}){\left(\frac{\pi }{L}\right)}^{4}}{9P_{e}-P}$$

Since $P_{e}-P>0$, the last term in Equation (31) can be neglected comparing with the first two terms. Following the procedure in Section 2, one obtains the maximum moment of the strut to be

(32) $$M_{max}=\frac{w_{I}P_{e}P}{P_{e}-P}+\frac{{\frac{1}{4}E}_{a}I{\left(\frac{w_{I}P_{e}}{P_{e}-P}\right)}^{3}(\frac{P}{P_{e}}){\left(\frac{\pi }{L}\right)}^{4}P}{P_{e}-P}$$

And

(33) $$\sigma_{max}=\frac{P}{A}+\left(\frac{w_{I}P_{e}P}{P_{e}-P}+\frac{{\frac{1}{4}E}_{a}I{\left(\frac{w_{I}P_{e}}{P_{e}-P}\right)}^{3}(\frac{P}{P_{e}}){\left(\frac{\pi }{L}\right)}^{4}P}{P_{e}-P}\right)\frac{c}{I}$$

Write $P_{ea}=\frac{{E_{a}I\pi }^{2}}{L^{2}}$,

(34) $$\sigma_{max}=\frac{P}{A}+\frac{P}{P_{e}-P}\frac{\kappa kP_{e}}{A}\lambda +\frac{1}{4}{\left(\frac{P_{e}}{P_{e}-P}\right)}^{3}(\frac{P}{P_{e}})\frac{P}{\left(P_{e}-P\right)}\frac{P_{ea}}{A}\kappa k^{3}\pi^{2}\lambda$$

Denote $\sigma_{ea}=\frac{P_{ea}}{A}$, $\eta =\kappa k^{3}\pi^{2}/4$ and ε=κk to obtain the following:

(35) $$\sigma_{max}=\sigma +\frac{\sigma \sigma_{e}}{\sigma_{e}-\sigma }\varepsilon \lambda +\frac{{\left(\sigma_{e}\right)}^{2}\sigma^{2}\sigma_{ea}}{{\left(\sigma_{e}-\sigma \right)}^{4}}\eta \lambda$$

**Figure 3:**
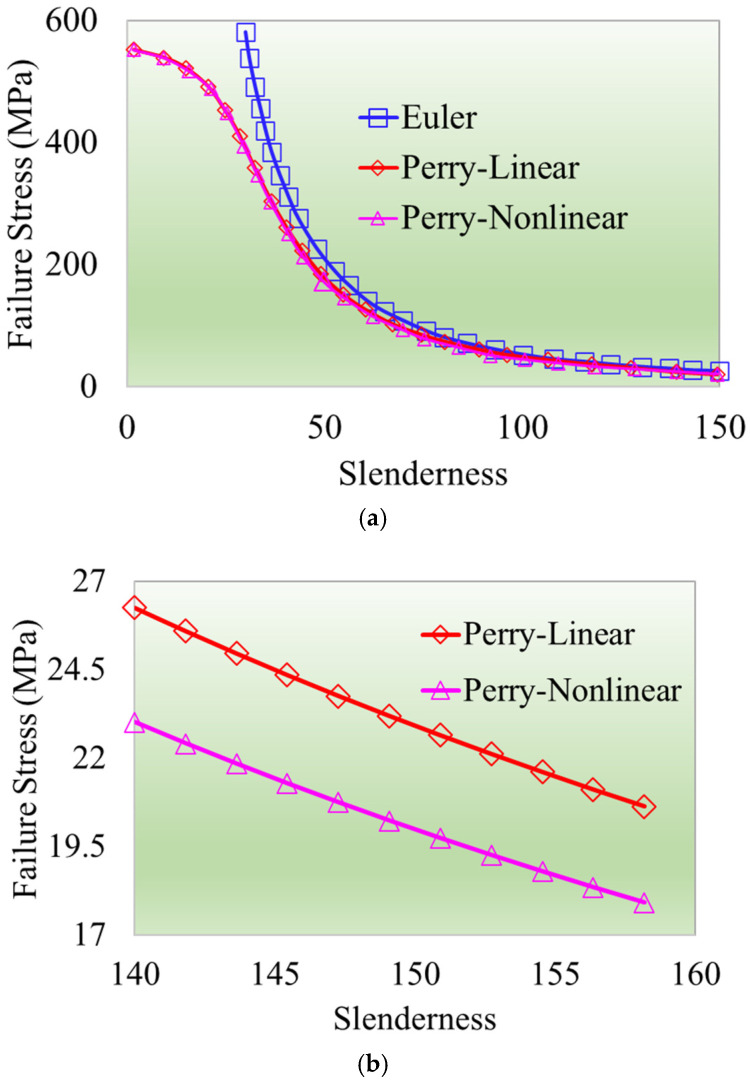
(**a**) Curves of Euler load, improved Perry’s load with/without material nonlinearity, and (**b**) effect of material nonlinearity at high slenderness.

Equation (35) is a 5th order polynomial and can be solved numerically. ${\;E}_{a}$, defined in Equation (15), can be estimated using available data from literature. A nonlinear relationship between fiber misalignment and modulus/stiffness has been reported. Since the present formulation is based on the small-slope assumption, $E_{a}$ is intended to characterize the initial stiffness degradation associated with small fiber misalignment rather than to fit the complete orientation-dependent modulus curve. A fiber misalignment of about 5° results in a stiffness reduction in the order of 10%, leading to $\frac{E_{a}}{E_{0}}>13$. Therefore, $E_{a}=14E_{0}$ is adopted in the present study. The failure stresses at various slenderness, with and without considering the effect of material nonlinearity, are then calculated and plotted in Figure 3. Note that predictions provided by the new Perry–Robertson formulation, derived in session 2, were re-denoted as Perry-Linear. In contrast, results obtained by considering the effect of material non-linearity were denoted as Perry-Nonlinear in Figure 3.

The case shown in Figure 3 uses $E_{0}=53.5\;GPa$ based on previous test data, and then, $E_{a}$ becomes 749 GPa. The difference between linear and nonlinear analysis is negligible for a strut of small slenderness. It goes up to around 13% when slenderness reaches 150.

## 4. Compression Tests

To validate the developed theory, a comprehensive test plan was designed and conducted. The plan includes material-level uniaxial compression tests to obtain the basic mechanical properties of the pultruded GFRP composite. Part-level compression buckling tests were then performed, considering various slenderness ratios ranging from 32 to 150, two production types (GFRP tubes and bars), and one supporting condition (simply supported). The data obtained from these tests were used to calibrate the developed model. Additionally, more part-level instability tests were conducted at different supporting conditions and slenderness ratios to further validate the accuracy and generalization ability of the proposed model. It is worth noting that a commonly used fiber volume fraction of 60% for pultruded GFRP insulators was considered throughout the experiments.

### 4.1. Material-Level Uniaxial Compression Test

**Figure 4:**
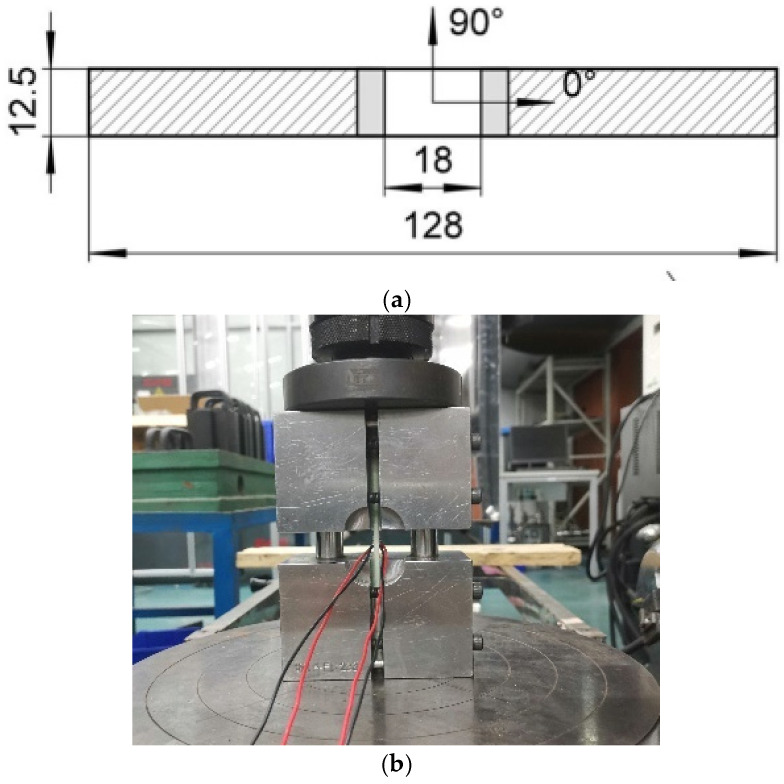
(**a**) The geometry of the uniaxial compression sample; (**b**) the experiment setup (unit: mm).

Samples used for compression tests were designed in accordance with the ASTM 6641 standard [28] in a sheet form, as shown in Figure 4a. The samples were machined from the pultruded bars and then glued with GFRP tabs at both ends. Strain gauges were attached on both sides to record the deformation and strain. Uniaxial compression tests (UCT) were performed using an MTS servo hydraulic testing system with a customized anti-buckling fixture (Figure 4b) at a constant test rate of 1.4 mm/min. Six replicates were tested to consider effect of material and test scatter.

**Figure 5:**
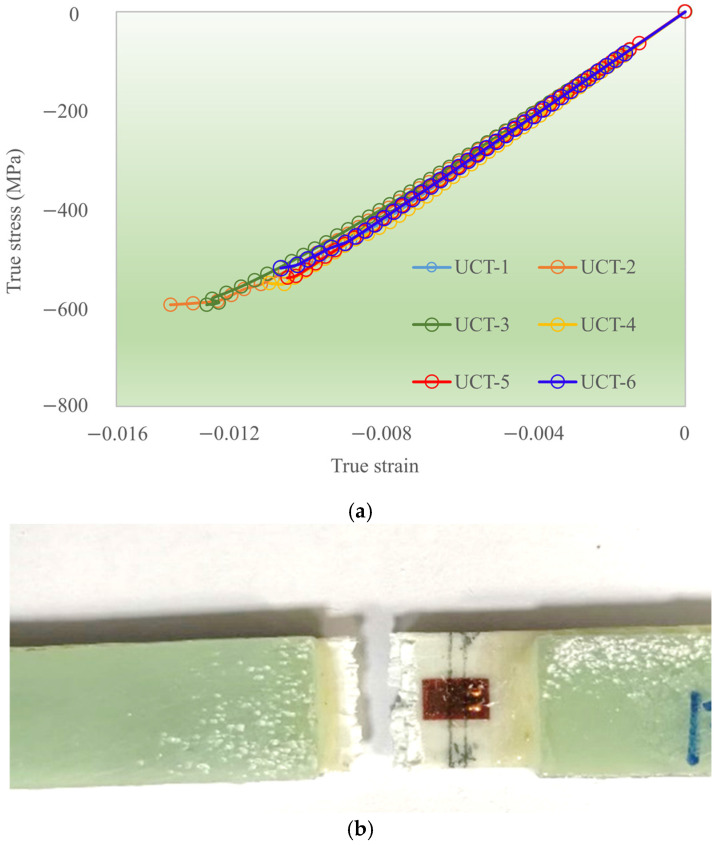
(**a**) The stress–strain curves of samples after compression; (**b**) the failed sample.

True stress vs. true strain curves are illustrated in Figure 5a and a failed specimen is shown in Figure 5b. As shown in Figure 5a, a good consistency based on the stress–strain curve was observed, indicating a well-controlled pultrusion process and compression test procedure. Mechanical properties such as the elastic modulus and ultimate compress strength (UCS) were tabulated in Table 1. Note that the average value of the elastic modulus was 53.6 GPa and agreed well with the previous result. The coefficients of variation obtained in this study were 6.1% for UCS and 2.3% for the elastic modulus. These values are comparable to, or even lower than, those reported for pultruded GFRP materials in the literature, where coefficients of variation for the strength and modulus are typically within the range of 2–10%, depending on the loading condition and fiber orientation [29]. Therefore, the scatter observed in the present study is considered typical and indicates good repeatability of the experimental results.

### 4.2. Part Level Compression Buckling Tests

**Figure 6:**
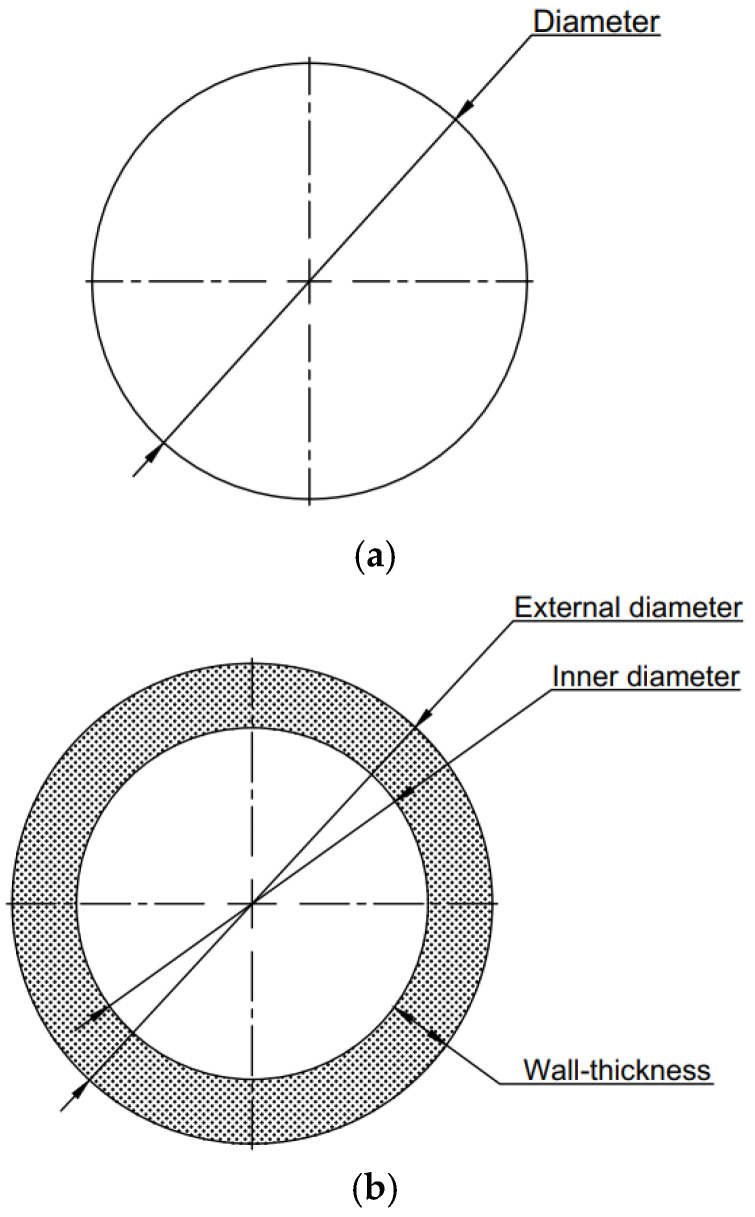
Cross-section view of pultruded parts for compression buckling test. (**a**) Solid struts; (**b**) hollowed tube.

Part-level compression buckling tests were performed to obtain the critical compression load and stress. Solid struts and hollowed tubes with various diameters and lengths (could also be unified as slenderness) were tested. A geometric illustration of pultruded parts is shown in Figure 6, and part dimensions are tabulated in Table 2, Table 3, Table 4 and Table 5.

**Figure 7:**
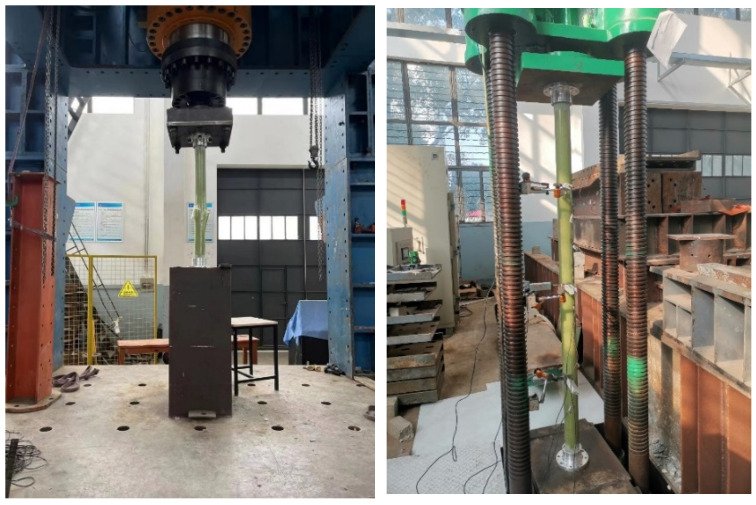
Hydraulic compression machine for the compression buckling test.

Solid struts with two diameters, i.e., ∅110 mm and ∅90 mm, respectively, and hollowed tubes with two diameters, i.e., ∅130–147 mm and ∅160–180 mm, were considered for testing, as shown in the above tables. Samples were cut to various lengths to take the effect of slenderness into account. As the critical compression load varied with sample geometry, two test frames with a load capacity of 1000 Ton and 200 Tons were employed to ensure test capacity and accuracy, as shown in Figure 7. The accuracy of load cells for both test frames was 1%, and travel ranges of the actuator were ±250 mm and ±200 mm respectively. The 1000-ton and 200-ton testing frames were used exclusively for large- and small-diameter specimens, respectively, and both systems were routinely calibrated. Since no specimen series was tested on both machines, inter-machine variability was not considered a source of uncertainty in the present study. As no dedicated standard is available for component-level buckling tests of pultruded GFRP insulator struts, the test procedure adopted in the present study was developed based on the general principles of axial compression testing and practical service conditions. Special attention was paid to specimen alignment and centering to ensure concentric load application and minimize eccentricity. The deflection of the tested part during the compression test was measured continuously by using a laser displacement sensor. Three replicates were tested for each configuration shown in Table 2, Table 3, Table 4 and Table 5, and an averaged critical compression load was then obtained for each configuration.

**Figure 8:**
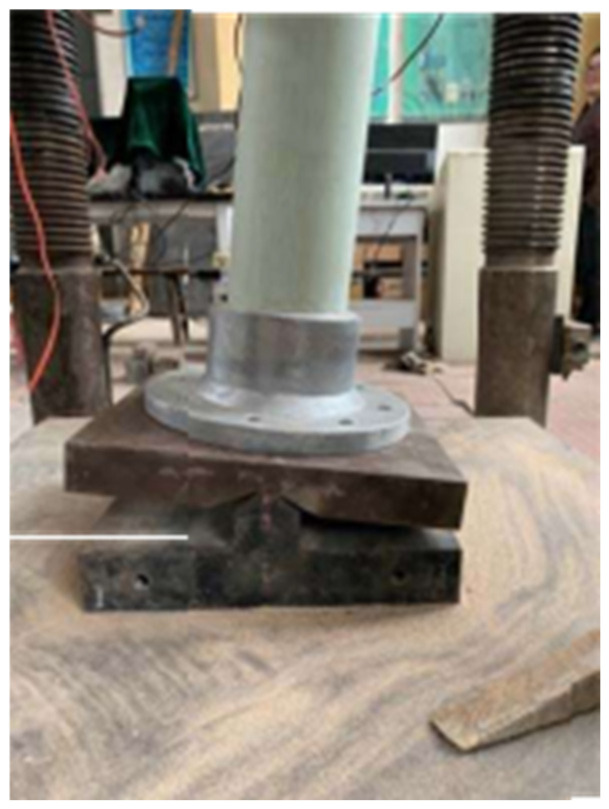
Simply support condition revealed by a V-shaped wedge.

As the supporting condition has a tremendous effect on the critical compression load, different supporting conditions were considered in this study. For the samples shown in Table 2 and Table 3, a simply supported constraint, revealed by a V-shaped wedge (Figure 8), was used at both ends, while for samples shown in Table 4 and Table 5, other boundary conditions such as clamped were also considered.

**Figure 9:**
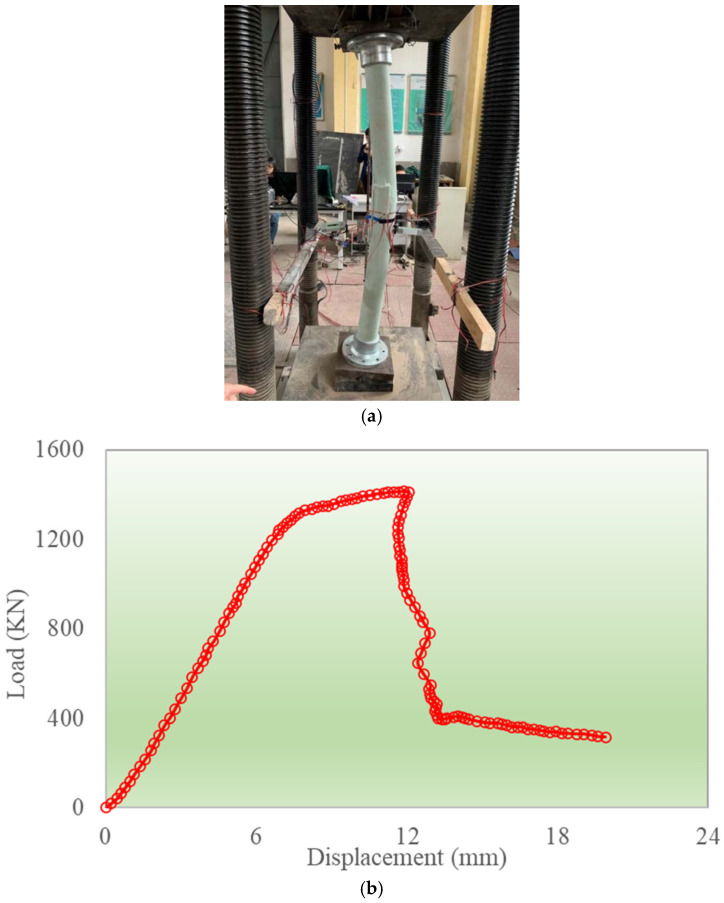
Compression test result for test set ∅110-1; (**a**) test set up, (**b**) load–displacement curve.

**Figure 10:**
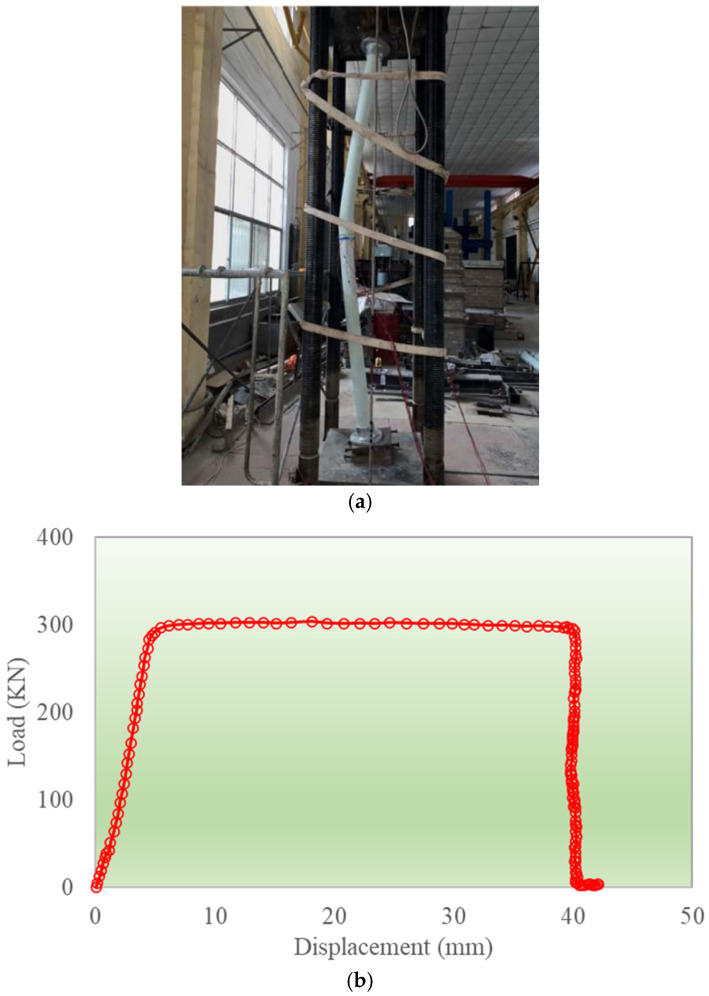
Compression test result for test set ∅110-5; (**a**) test set up, (**b**) load–displacement curve.

Part-level compression test results are listed in Table 6, Table 7, Table 8 and Table 9. Note that in the present study, the critical compression load was defined as the peak load attained during the test. It is not surprising to see that the critical compression load varied with the sample size and slenderness. Moreover, when the slenderness is relatively low, e.g., solid strut with a diameter of ∅110 mm and a length of 1850 mm (a slenderness of 57), compression failure is the most frequently observed failure mode. A failed specimen and the corresponding load–displacement curve are shown in Figure 9a,b. For this failure mode, an abrupt turn could be observed on the load–displacement curve, indicating the onset of material failure. In contrast, when the slenderness is relatively high, one sees the bending of the sample very clearly before material failure, as shown in Figure 10a for a solid strut with a diameter of ∅110 mm and a length of 3380 mm (a slenderness of 123). The corresponding load–displacement curve is shown in Figure 10b, which has a period of increment of deflection with a limited increment in the load, indicating an unstable state associated with buckling. Therefore, the present test program intentionally covers both material-dominated failures at low slenderness and instability-dominated failures at high slenderness, providing a representative basis for subsequent model calibration and validation. Such a broad range of failure mechanisms enables the developed model to realistically capture the transition from material-controlled behavior to buckling-controlled behavior over a wide range of slenderness ratios encountered in practical structures.

Test results shown in Table 6 and Table 7 were used exclusively for model calibration, whereas an independent dataset consisting of the test results shown in Table 8 and Table 9 was employed for validation. This separation was adopted to assess the predictive accuracy and generalization capability of the developed model and to avoid overfitting during parameter calibration. 

## 5. Model Calibration and Validation

The proposed model was then calibrated by using test data obtained hereinbefore. The parameter to be calibrated was the initial imperfection *k* defined in Equation (13). The calibration was achieved using the solver function in Microsoft Excel. The fitting error was defined as the difference in model prediction and test measurement, normalized by the test measurement. Accordingly, the objective function used for calibration can be expressed as

(36) $$Obj=\sum_{i=1}^{N}{\left(\frac{P_{pred,i}-P_{test,i}}{P_{test,i}}\right)}^{2}$$

where $P_{pred,i}$ and $P_{test,i}$ are the predicted and measured critical loads, respectively, and *N* is the number of calibration samples. The model parameter was determined by minimizing the objective function defined in Equation (36).

**Figure 11:**
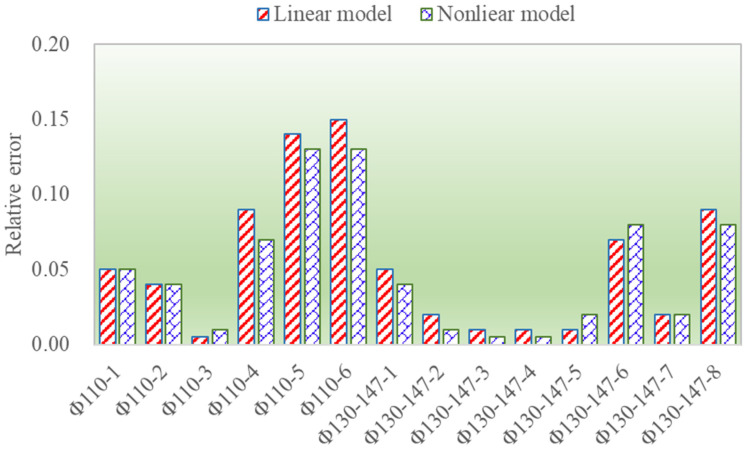
Relative error between test data and predictions.

For the linear model, the optimized imperfection fitting was k=0.0031, and the corresponding summation of the square of the error was 0.072. For the nonlinear model, k=0.0029, with the summation of square of error being 0.051. The relative errors between test data and predictions of both models have been illustrated in Figure 11. Apparently, predictions agreed well with test data by using both models, and the nonlinear model could further improve the accuracy.

The above predictions assume that initial imperfection is linearly related to the length of the strut/tube (Equation (13)), which ignored the effect of the sample diameter. A more generalized assumption could be given as

(37) $$\omega_{I}=(k_{0}+k_{1}\lambda )L$$

where λ is slenderness and $k_{0}$ and $k_{1}$ are empirical constants that need to be calibrated.

**Figure 12:**
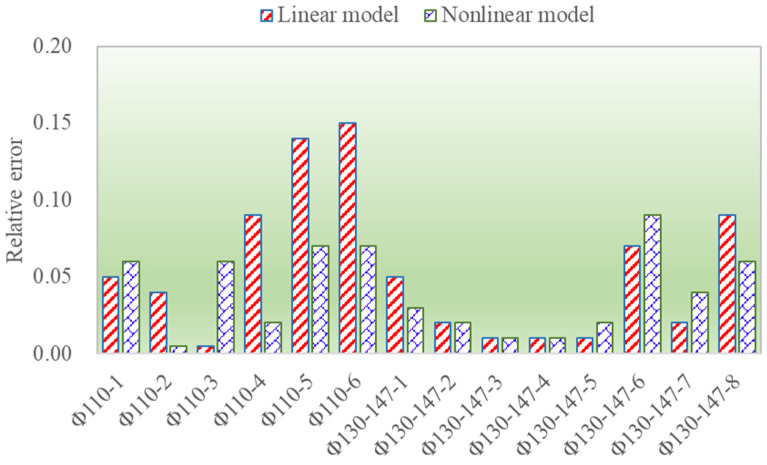
Relative error between test data and predictions by using the initial imperfection relation defined in Equation (35).

By using the same optimization procedure in Excel, one can get the improved fitting as shown in Figure 12. In this case, for linear Perry–Robertson, $k_{0}=0,$ $k_{1}=0.0000644$, and the square error is 0.0372; for the nonlinear case, $k_{0}=0$, $k_{1}=0.0000583$, and the square error is 0.0321. It is noted that, for the nonlinear formulation, all predicted buckling loads differ from the corresponding test measurements by less than 10%. Furthermore, the average relative error and standard deviation are 4.0% and 2.7%, respectively, while the maximum relative error is 9.1%, indicating good agreement between the model predictions and the experimental results. Considering that the tested samples have a wide variation with different slenderness and including both solid and circular cross-sections, the good prediction is encouraging.

The proposed model was further validated by using new test data sets (i.e., the sample configurations and test results shown in Table 8 and Table 9). Predictions and relative errors are shown in Table 10. Note that $k_{0}$ and $k_{1}$ obtained above were directly adapted, and only predictions for the nonlinear model were presented due to its’ higher accuracy. As shown in Table 10, the relative errors for four cases are all within 15% despite of the geometry and boundary condition, which further proves the accuracy and generalization ability of proposed model.

## 6. Conclusions

In this paper, the material properties of pultruded glass-fiber-reinforced epoxy composites were characterized, and the stability behavior of composite struts under axial compression was investigated. Based on the pressure-sensitive stability theory, an extended Perry–Robertson formulation considering initial imperfections was developed. In addition, the geometric nonlinearity arising from the change in fiber orientation with respect to the loading direction during buckling was incorporated into the formulation. The main conclusions are summarized as follows:

(1) The buckling behavior of pultruded GFRP struts was analyzed based on Perry–Robertson’s assumption that a strut with initial imperfection fails when the maximum stress reaches the ultimate material strength. By relating the initial imperfection directly to the strut length, the buckling load can be expressed explicitly as a function of slenderness. The present formulation provides a smooth transition between material-controlled failure and buckling-controlled failure over the range of slenderness ratios investigated in the present study.
(2) Material nonlinearity associated with the change in fiber orientation during buckling was incorporated into the Perry–Robertson framework, resulting in an analytical nonlinear buckling formulation. A comparison with the calibration dataset indicates that the nonlinear formulation provides improved agreement with test measurements compared with its linear counterpart, particularly for members with relatively large slenderness ratios where material nonlinearity becomes more pronounced. More significant improvements are obtained when the imperfection is assumed to vary nonlinearly with slenderness.
(3) Part-level compression tests covering both material-dominated failures at low slenderness and instability-dominated failures at high slenderness were conducted for model calibration and independent validation. For the validation dataset summarized in Table 10, the prediction error of the nonlinear formulation was generally within 15%, indicating satisfactory agreement with experimental measurements for the range of geometries and support conditions investigated in the present study.
(4) The present formulation relies on simplified assumptions regarding initial imperfections, and the perturbation analysis was truncated after the first-order term. Moreover, the model parameters were calibrated using the current experimental dataset. Therefore, further studies are required to assess the applicability of the model to other pultruded GFRP geometries, material systems, boundary conditions, and loading configurations.

## Figures and Tables

**Table 1:** The uniaxial compression results of GFRP.

	UCT-1	UCT-2	UCT-3	UCT-4	UCT-5	UCT-6	AVE	SDV	COV (%)
UCS (MPa)	517	596	589	554	542	519	552.8	33.82	6.1
E (GPa)	54.2	52.9	52.4	55.9	53.3	53.4	53.6	1.2	2.3

**Table 2:** Dimension and support condition of solid struts with diameter ∅110.

Set ID	Diameter (mm)	Length (mm)	Slenderness	Support Condition
∅110-1	110	1580	57	Simply Supported
∅110-2	110	1980	72	Simply Supported
∅110-3	110	2380	87	Simply Supported
∅110-4	110	2580	101	Simply Supported
∅110-5	110	3180	116	Simply Supported
∅110-6	110	3380	123	Simply Supported

**Table 3:** Dimension and support condition of hollowed tubes with diameter ∅130–147.

Set ID	Inner Diameter (mm)	External Diameter (mm)	Length (mm)	Slenderness	Support Condition
∅130–147-1	130	147	1578	32	Simply supported
∅130–147-2	130	147	1978	40	Simply supported
∅130–147-3	130	147	2178	44	Simply supported
∅130–147-4	130	147	2378	48	Simply supported
∅130–147-5	130	147	2578	53	Simply supported
∅130–147-6	130	147	2778	57	Simply supported
∅130–147-7	130	147	2878	61	Simply supported
∅130–147-8	130	147	3378	69	Simply supported

**Table 4:** Dimension of and support condition of solid struts with diameter ∅90.

Set ID	Diameter (mm)	Length (mm)	Slenderness	Support Condition
∅90-1	90	1100	48.89	Clamped
∅90-2	90	2200	97.78	Clamped
∅90-3	90	3300	146.67	Clamped + Simply supported
∅90-4	90	3300	146.67	Clamped

**Table 5:** Dimension and support condition of hollowed tubes solid struts with diameter ∅160–180.

Set ID	Inner Diameter (mm)	External Diameter (mm)	Length (mm)	Slenderness	Support Condition
∅160–180	160	180	6000	99.65	Clamped + Simply supported

**Table 6:** Test results for solid struts with diameter ∅110.

Set-ID	Failure Load (KN)	Failure Load (KN)	Failure Load (KN)	Average Failure Load (KN)	Average Failure Stress (MPa)
∅110-1	1418	1378	1387	1394	147
∅110-2	828	895	800	841	88
∅110-3	606	629	621	619	65
∅110-4	526	480	492	499	53
∅110-5	300	315	297	304	32
∅110-6	257	303	245	268	28

**Table 7:** Test results for hollowed tubes with diameter ∅130–147.

Set ID	Failure Load (KN)	Failure Load (KN)	Failure Load (KN)	Average Failure Load (KN)	Average Failure Stress (MPa)
∅130–147-1	1401	1343	1481	1408	381
∅130–147-2	1004	1020	989	1004	272
∅130–147-3	861	880	813	851	230
∅130–147-4	700	721	750	724	196
∅130–147-5	643	619	652	638	173
∅130–147-6	588	592	669	616	167
∅130–147-7	498	457	519	491	133
∅130–147-8	355	349	336	347	94

**Table 8:** Test results for solid struts with diameter ∅90.

Set ID	Failure Load (KN)	Failure Load (KN)	Failure Load (KN)	Average Failure Load (KN)	Average Failure Stress (MPa)
∅90-1	3602	3585	3301	3496	550
∅90-2	1430	1352	1395	1392	219
∅90-3	316	305	-	311	49
∅90-4	567	567	557	564	89

**Table 9:** Test results for hollowed tubes with diameter ∅160–180.

Set ID	Failure Load (KN)	Failure Load (KN)	Failure Load (KN)	Average Failure Load (KN)	Average Failure Stress (MPa)
∅160–180	488.7	440.6	448.4	459	86

**Table 10:** Model validation by using new test data and different supporting conditions.

Set ID	Diameter (mm)	Length (mm)	Support Condition	Test Data (KN)	Prediction (KN)	Relative Error
∅90-1	90	1100	Clamped	3495	3048	0.13
∅90-2	90	2200	Clamped	1392	1191	0.14
∅90-3	90	3300	Clamped + Simply supported	311	273	0.12
∅90-4	90	3300	Clamped	564	539	0.04
∅160–180	160/180	6000	Clamped + Simply supported	459	512	0.11

## Data Availability

The original contributions presented in this study are included in the article. Further inquiries can be directed to the corresponding author.
